# Editorial: Reducing healthcare-associated infections through antimicrobial materials

**DOI:** 10.3389/fcimb.2024.1446870

**Published:** 2024-07-09

**Authors:** Sarah Ghamrawi, Lei Ren

**Affiliations:** ^1^ 1Host-Pathogen Interaction Study Group (GEIHP), University Hospital of Angers, Angers, France; ^2^ Melanoma Institute Australia, Sydney, NSW, Australia; ^3^ Department of Biomaterials, State Key Lab of Physical Chemistry of Solid Surface, College of Materials, Xiamen University, Xiamen, China

**Keywords:** antimicrobial, catheter associated urinary tract infection (CAUTI), biofilm, central venous catheter (CVC), carbon dot (CD), textile, healthcare associated infection (HAI), iron chelator

Healthcare-associated infections (HAIs) represent a persistent challenge in healthcare systems worldwide, contributing to increased morbidity, mortality, and healthcare costs.

According to the most recent report by the World Health Organisation (WHO, 2022), between 7% and 15% of patients in acute-care hospitals worldwide are estimated to develop at least one healthcare-associated infection (HAI) during their hospitalization. Therefore having systems in place for the surveillance of HAIs, monitoring infection rates, identifying trends, implementing interventions to prevent future infections and publishing those results helps evaluate those actions in different countries (Allerberger and Küenburg, 2019; Russo et al., 2019; Alrebish et al., 2022; Antimicrobial Resistance Collaborators, 2022; Hu et al., 2022; Thandar et al., 2022; Bansal et al., 2023; CDC, 2023).

The continuous innovation in biosafety strategies and materials helps reduce the occurrence and severity of HAIs for an increasing number of sick patients. (Ghamrawi et al., 2017; Ghamrawi et al., 2020; Li et al., 2021; Li et al., 2022). In this editorial, we explore recent research efforts aimed at mitigating the risk of HAIs through diverse and innovative approaches. The articles presented in this editorial converge on the overarching goal of enhancing biosafety in different healthcare settings through multifaceted strategies:

Iron is essential for the growth and proliferation of pathogenic microorganisms and the development of biofilms. Sequestration of iron by iron chelators and combining iron chelators with antibacterial and antifungal agents presents an interesting avenue for controlling central venous catheter-associated infections. Itoh et al. discusses approaches to incorporate iron chelators into antimicrobial-impregnated central venous catheters to further suppress microbial colonization and biofilm formation in long term central venous catheters. These approaches hold potential for reducing the incidence of catheter-related infections and improving patient outcomes.

Catheter-Associated Urinary Tract Infections (CAUTI) in healthcare settings is a significant challenge for the use of urinary catheters. The continuous flow of urine through these devices promotes biofilm formation that could turn into a source of infection to the patient difficult to combat because of reduced immune responses and drug efficacy in these areas. Rajaramon et al. reviewed emerging evidence-based approaches to control CAUTI, focusing on modifying the surface properties of urinary catheters to prevent biofilm formation and inhibit microbial growth. By incorporating anti-fouling, biocidal, and anti-adhesive properties into urinary catheter surfaces, these strategies offer promising solutions for managing CAUTI and reducing associated morbidity and mortality. The paper emphasizes the importance of continuous innovation in catheter design to mitigate the risks associated with prolonged catheter use.

Carbon dots (CDs) have emerged as innovative carriers for antimicrobial agents with potential applications in combating oral microbial infections. Traditional treatments using antibiotics face challenges such as microbial resistance, drug blocking by biofilms, and drug dilution in the oral environment, making them less effective. Jiang et al. reviewed the antimicrobial efficacy of CDs against diverse oral pathogens and highlighted their utility in microbial imaging and detection. CDs offer a promising avenue for developing novel strategies to prevent and treat oral infections, addressing the limitations of traditional antibiotic treatments and discusses current challenges for the development of carbon dots and avenues to overcome these limitations. CDs are highlighted for their excellent biophysical and chemical properties, including low toxicity, stability, and strong antimicrobial abilities against various oral pathogens like planktonic bacteria, intracellular bacteria, and fungi. The review also explores CDs’ capabilities in microbial imaging and early diagnosis of oral infections, suggesting that CDs can significantly enhance clinical treatment options by providing both detection and therapeutic functions. The authors discuss the future prospects and current limitations of CDs in clinical applications, emphasizing the need for further research to fully realize their potential in combating oral microbial infections.

Textiles impregnated with antimicrobial substances have emerged as one of the biosafety strategies in healthcare services. Schneider et al. conducted a systematic review of intervention studies to evaluate the efficacy of antimicrobial textiles in reducing microbial load and healthcare-associated infection (HAI) rates. The study reviews 23 intervention studies, finding that textiles impregnated with substances like copper, silver, zinc oxide, titanium, and quaternary ammonium compounds are effective in lowering microbial contamination when used by patients, healthcare professionals, and in healthcare environments. The review highlights significant biosafety benefits, emphasizing the potential of antimicrobial textiles to minimize infection risks in healthcare settings. However, it also notes concerns regarding microbial resistance and the need for cautious implementation. The study contributes valuable insights for the safe and effective use of antimicrobial textiles to enhance infection control in healthcare services.

The need for effective prevention of transmission became more evident with the COVID-19 pandemic. Chaki et al. developed a validation method for sample inactivation to ensure safe transportation of SARS-CoV-2 from high containment laboratories. This method is particularly useful when prioritizing sample innocuity over sample integrity. The reliable inactivation of pathogens before they enter healthcare environments supports the overall effort to control and mitigate the spread of infections, safeguarding both patients and healthcare workers.

Finally, This editorial highlights the need for ongoing research, collaboration, and innovation in addressing the complex challenges posed by HAIs, ultimately aiming to create a safer and more sustainable healthcare environment for all. While innovative biosafety strategies and materials hold immense potential in mitigating the risk of HAIs, challenges persist in their implementation and widespread adoption. Factors such as cost-effectiveness, sustainability, and antimicrobial resistance necessitate careful consideration in the development and deployment of these interventions. While Infection Prevention and Control programs by WHO (Allegranzi, 2016) as well as guidance provided by the Centre for Disease and Control (CDC, 2024) provide excellent guides and the gold standards for ongoing efforts to reduce HAIs, however, by embracing interdisciplinary approaches and fostering a culture of innovation, healthcare organizations will further mitigate the risk and burden of HAIs, and advance towards safer and more resilient healthcare delivery and safeguard the patient’s well-being.

## Author contributions

SG: Conceptualization, Writing – original draft, Writing – review & editing. LR: Writing – review & editing.

## References

[B1] Allegranzi B. (2016).“Health Care without Avoidable Infections The Critical Role of Infection Prevention and Control,”. In: . Available online at: https://iris.who.int/bitstream/handle/10665/246235/WHO-HIS-SDS-2016.10-eng.pdf?sequence=1.

[B2] Allerberger F. Küenburg B. (2019). Organization of control of nosocomial infections in central Eastern European countries. Wiener Medizinische Wochenschrift 169, 1–2. doi: 10.1007/s10354-018-0671-x PMC6373268 30725366

[B3] Alrebish S. A. Yusufoglu H. S. Alotibi R. F. Abdulkhalik N. S. Ahmed N. J. Khan A. H. (2022). Epidemiology of healthcare-associated infections and adherence to the HAI prevention strategies. Healthcare 11, 635. doi: 10.3390/healthcare11010063 PMC9818953 36611523

[B4] Antimicrobial Resistance Collaborators (2022). Global mortality associated with 33 bacterial pathogens in 2019: A systematic analysis for the global burden of disease study 2019. Lancet (London England) 400, 2221–2248. doi: 10.1016/S0140-6736(22)02185-7 36423648 PMC9763654

[B5] Bansal N. Goyal P. Basu D. Batra U. Sachdeva N. Joga S.. (2023). Impact of improving infection control and antibiotic stewardship practices on nosocomial infections and antimicrobial resistance in an oncology centre from India. Indian J. Med. Microbiol. 45, 100383. doi: 10.1016/j.ijmmb.2023.100383 37573060

[B6] CDC. (2023). “Centers for disease control and prevention (CDC), 2023,” in Current HAI progress report (CDC: Centers for Disease Control and Prevention). Available at: https://www.cdc.gov/hai/data/portal/progress-report.html.

[B7] CDC. (2024). “HAI prevention and control for healthcare,” in Healthcare-associated infections (HAIs). (CDC: Centers for Disease Control and Prevention). Available at: https://www.cdc.gov/healthcare-associated-infections/hcp/prevention-healthcare/index.html.

[B8] Ghamrawi S. Bouchara J.-P. Corbin A. Rogalsky S. Tarasyuk O. Bardeau J.-F.. (2022). Inhibition of fungal growth by silicones modified with cationic biocides. 22, 100716. doi: 10.1016/j.mtcomm.2019.100716

[B9] Ghamrawi S. Bouchara J.-P. Tarasyuk O. Rogalsky S. Lyoshina L. Bulko O.. (2017). Promising silicones modified with cationic biocides for the development of antimicrobial medical devices. Materials Science & Engineering. C, Materials for Biological Applications. 75, 969–979. doi: 10.1016/j.msec.2017.03.013 28415553

[B10] Hu F. Yuan L. Yang Y. Xu Y. Huang Y. Hu Y.. (2022). A multicenter investigation of 2,773 cases of bloodstream infections based on China antimicrobial surveillance network (CHINET). Front. Cell. Infection Microbiol. 12. doi: 10.3389/fcimb.2022.1075185 PMC9798236 36590586

[B11] Li W. Eng San T. Miao W. Zuyong W. Lei R. (2021). Surface design for antibacterial materials: from fundamentals to advanced strategies. Adv. Sci. (Weinheim, Baden-Wurttemberg, Germany) 8 (19), e2100368. doi: 10.1002/advs.202100368 PMC8498904 34351704

[B12] Li W. Guanping H. Jingfeng C. Yaming Z. Xi Z. Miao W.. (2022). Multi-stimulus responsive multilayer coating for treatment of device-associated infections. J. Funct. Biomater. 13 (1), 24. doi: 10.3390/jfb13010024 35323224 PMC8954600

[B13] Russo P. L. Stewardson A. J. Cheng A. C. Bucknall T. Mitchell B. G. (2019). The prevalence of healthcare associated infections among adult inpatients at nineteen large australian acute-care public hospitals: a point prevalence survey. Antimicrobial Resistance Infection Control 8, 1145. doi: 10.1186/s13756-019-0570-y PMC6628491 31338161

[B14] Thandar M. M. Rahman M. O. Haruyama R. Matsuoka S. Okawa S. Moriyama J.. (2022). Effectiveness of infection control teams in reducing healthcare-associated infections: A systematic review and meta-analysis. Int. J. Environ. Res. Public Health 19, 17075. doi: 10.3390/ijerph192417075 36554953 PMC9779570

[B15] World Health Organisation. (2022). WHO Launches First Ever Global Report on Infection Prevention and Control. Available online at: https://www.who.int/news/item/06-05-2022-who-launches-first-ever-global-report-on-infection-prevention-and-control.

